# Supplementary material for: Discovery and pharmacophoric characterization of chemokine network inhibitors using phage-display, saturation mutagenesis and computational modelling
Source: Nat Commun. 2023 Sep 16;14:5763. doi: 10.1038/s41467-023-41488-z (PMC10505172; doi:10.1038/s41467-023-41488-z)
Supplement: Supplementary file 4 — Source Data [file 41467_2023_41488_MOESM4_ESM.zip › SourceData/scripts/xNNKs_20230808183658.tar!/xNNKs/analysis/SuppTable5.docx]

Supplementary Table 5: Exact P values for phage-display mutagenesis analyses of
scanning mutants, comparisons to HD2 as negative control

| Residue | Substitution type | Number of samples | P value |
| --- | --- | --- | --- |
| E2 | alanine | 24 | 8.42e-03 |
| D3 | alanine | 24 | 2.84e-05 |
| D4 | alanine | 24 | 1.27e-10 |
| Y5 | alanine | 24 | 6.36e-11 |
| Y8 | alanine | 24 | 2.65e-11 |
| P10 | alanine | 24 | 1.05e-13 |
| L11 | alanine | 24 | 1.71e-02 |
| T12 | alanine | 24 | 1.95e-03 |
| C13 | alanine | 24 | 6.66e-16 |
| Y14 | alanine | 24 | 2.44e-11 |
| Y5 | anionic | 48 | 4.29e-04 |
| T6 | anionic | 48 | 5.00e-05 |
| A7 | anionic | 48 | 3.77e-02 |
| Y8 | anionic | 48 | 4.12e-11 |
| A9 | anionic | 48 | 2.49e-06 |
| P10 | anionic | 48 | 3.80e-09 |
| L11 | anionic | 48 | 1.93e-13 |
| T12 | anionic | 48 | 1.94e-07 |
| C13 | anionic | 48 | 0.00e+00 |
| Y14 | anionic | 48 | 1.18e-11 |
| F15 | anionic | 48 | 2.74e-05 |
| E1 | arginine | 24 | 8.89e-11 |
| E2 | arginine | 24 | 3.01e-09 |
| D3 | arginine | 24 | 7.31e-14 |
| D4 | arginine | 24 | 3.08e-13 |
| Y5 | arginine | 24 | 2.44e-15 |
| Y8 | arginine | 24 | 0.00e+00 |
| A9 | arginine | 24 | 6.46e-06 |
| P10 | arginine | 24 | 0.00e+00 |
| T12 | arginine | 24 | 1.60e-04 |
| C13 | arginine | 24 | 0.00e+00 |
| Y14 | arginine | 24 | 0.00e+00 |
| F15 | arginine | 24 | 7.32e-03 |
| T16 | arginine | 24 | 1.77e-05 |
| Y5 | aspartate | 24 | 9.15e-08 |
| T6 | aspartate | 24 | 2.35e-05 |
| A7 | aspartate | 24 | 3.73e-02 |
| Y8 | aspartate | 24 | 5.38e-11 |
| A9 | aspartate | 24 | 0.00e+00 |
| P10 | aspartate | 24 | 1.02e-06 |
| L11 | aspartate | 24 | 2.66e-15 |
| T12 | aspartate | 24 | 5.65e-08 |
| C13 | aspartate | 24 | 1.11e-16 |
| Y14 | aspartate | 24 | 1.53e-09 |
| F15 | aspartate | 24 | 7.22e-06 |
| E1 | cationic | 48 | 2.83e-12 |
| E2 | cationic | 48 | 5.98e-10 |
| D3 | cationic | 48 | 6.03e-14 |
| D4 | cationic | 48 | 0.00e+00 |
| Y5 | cationic | 48 | 0.00e+00 |
| T6 | cationic | 48 | 3.35e-02 |
| Y8 | cationic | 48 | 0.00e+00 |
| A9 | cationic | 48 | 6.37e-11 |
| P10 | cationic | 48 | 0.00e+00 |
| L11 | cationic | 48 | 2.59e-02 |
| T12 | cationic | 48 | 4.13e-08 |
| C13 | cationic | 48 | 0.00e+00 |
| Y14 | cationic | 48 | 0.00e+00 |
| F15 | cationic | 48 | 2.59e-04 |
| T16 | cationic | 48 | 1.01e-04 |
| E1D | conservative | 24 | 4.81e-02 |
| A9G | conservative | 24 | 6.39e-12 |
| P10A | conservative | 24 | 6.66e-16 |
| T12A | conservative | 24 | 3.99e-04 |
| C13S | conservative | 24 | 2.27e-09 |
| T6 | glutamate | 24 | 2.31e-03 |
| Y8 | glutamate | 24 | 3.45e-09 |
| P10 | glutamate | 24 | 1.83e-08 |
| L11 | glutamate | 24 | 1.56e-07 |
| T12 | glutamate | 24 | 5.02e-05 |
| C13 | glutamate | 24 | 1.11e-13 |
| Y14 | glutamate | 24 | 9.90e-10 |
| F15 | glutamate | 24 | 1.62e-03 |
| E1 | hydrophobic | 96 | 1.25e-02 |
| E2 | hydrophobic | 96 | 3.17e-03 |
| D3 | hydrophobic | 96 | 1.13e-13 |
| D4 | hydrophobic | 96 | 1.11e-16 |
| Y5 | hydrophobic | 96 | 1.32e-10 |
| T6 | hydrophobic | 96 | 7.51e-04 |
| Y8 | hydrophobic | 96 | 0.00e+00 |
| A9 | hydrophobic | 96 | 0.00e+00 |
| P10 | hydrophobic | 96 | 0.00e+00 |
| L11 | hydrophobic | 72 | 7.94e-03 |
| C13 | hydrophobic | 96 | 1.11e-16 |
| Y14 | hydrophobic | 96 | 1.67e-10 |
| E2 | isoleucine | 24 | 4.23e-02 |
| D3 | isoleucine | 24 | 3.02e-09 |
| D4 | isoleucine | 24 | 1.78e-11 |
| Y5 | isoleucine | 24 | 3.93e-09 |
| T6 | isoleucine | 24 | 1.34e-09 |
| Y8 | isoleucine | 24 | 0.00e+00 |
| A9 | isoleucine | 24 | 0.00e+00 |
| P10 | isoleucine | 24 | 0.00e+00 |
| L11 | isoleucine | 24 | 3.01e-03 |
| C13 | isoleucine | 24 | 1.63e-10 |
| Y14 | isoleucine | 24 | 1.14e-04 |
| E2 | leucine | 24 | 3.36e-02 |
| D3 | leucine | 24 | 6.23e-08 |
| D4 | leucine | 24 | 1.53e-09 |
| Y5 | leucine | 24 | 1.08e-02 |
| Y8 | leucine | 24 | 5.96e-13 |
| A9 | leucine | 24 | 4.30e-09 |
| P10 | leucine | 24 | 1.33e-15 |
| C13 | leucine | 24 | 2.89e-15 |
| Y14 | leucine | 24 | 4.48e-09 |
| E1 | lysine | 24 | 8.39e-08 |
| E2 | lysine | 24 | 4.84e-06 |
| D3 | lysine | 24 | 1.61e-08 |
| D4 | lysine | 24 | 2.00e-15 |
| Y5 | lysine | 24 | 9.15e-11 |
| T6 | lysine | 24 | 2.08e-04 |
| Y8 | lysine | 24 | 1.06e-13 |
| A9 | lysine | 24 | 1.28e-10 |
| P10 | lysine | 24 | 0.00e+00 |
| L11 | lysine | 24 | 3.01e-03 |
| T12 | lysine | 24 | 1.31e-08 |
| C13 | lysine | 24 | 0.00e+00 |
| Y14 | lysine | 24 | 0.00e+00 |
| F15 | lysine | 24 | 3.45e-04 |
| T16 | lysine | 24 | 3.09e-02 |
| E2 | methionine | 24 | 6.20e-03 |
| D3 | methionine | 24 | 7.20e-07 |
| D4 | methionine | 24 | 1.09e-09 |
| Y5 | methionine | 24 | 5.72e-04 |
| Y8 | methionine | 24 | 9.57e-14 |
| A9 | methionine | 24 | 1.90e-05 |
| P10 | methionine | 24 | 2.00e-15 |
| C13 | methionine | 24 | 1.04e-10 |
| Y14 | methionine | 24 | 1.69e-06 |
| E1 | valine | 24 | 1.40e-03 |
| D3 | valine | 24 | 3.60e-11 |
| D4 | valine | 24 | 3.86e-13 |
| Y5 | valine | 24 | 5.68e-13 |
| T6 | valine | 24 | 2.50e-05 |
| Y8 | valine | 24 | 8.83e-09 |
| A9 | valine | 24 | 1.36e-13 |
| P10 | valine | 24 | 0.00e+00 |
| C13 | valine | 24 | 3.86e-07 |
| Y14 | valine | 24 | 6.92e-06 |
